# Editorial: Methods and application in cardiovascular and smooth muscle pharmacology: 2021

**DOI:** 10.3389/fphar.2022.1049022

**Published:** 2022-10-21

**Authors:** Ahmed F. El-Yazbi, Ali H. Eid, Fouad A. Zouein, Khaled S. Abd-Elrahman

**Affiliations:** ^1^ Department of Pharmacology and Toxicology, Faculty of Pharmacy, Alexandria University, Alexandria, Egypt; ^2^ Faculty of Pharmacy, Alamein International University, Al Alamein, Egypt; ^3^ Department of Basic Medical Sciences, College of Medicine, QU Health, Qatar University, Doha, Qatar; ^4^ Department of Pharmacology and Toxicology, Faculty of Medicine, American University of Beirut, Beirut, Lebanon; ^5^ Department of Signaling and Cardiovascular Pathophysiology, UMR-S 1180, Inserm, Université Paris-Saclay, Paris-Saclay, France; ^6^ The Cardiovascular Renal and Metabolic Diseases Research Center of Excellence, American University of Beirut Medical Center, Beirut, Lebanon; ^7^ Department of Pharmacology and Toxicology, School of Medicine, University of Mississippi Medical Center, Jackson, MS, United States; ^8^ Department of Cellular and Molecular Medicine, Faculty of Medicine, University of Ottawa, Ottawa, ON, Canada; ^9^ Department of Pharmacology and Therapeutics, College of Medicine and Health Sciences, Khalifa University, Abu Dhabi, United Arab Emirates

**Keywords:** cardiovasclar disease, biomarkers, natural products, biomedical research, disease outcome

Despite significant advances in basic, translational, and clinical research tackling heart disease, cardiovascular pathologies remain among the leading causes of mortality and morbidity worldwide, being responsible for one-third of global deaths as estimated by the WHO (Organization, 2021). The complexity of risk factors and pathways underlying the development of cardiovascular disorders (CVDs) limits the efficacy of a given therapeutic intervention and necessitates combined pharmacological approaches, as well as lifestyle modification to provide a reasonable health impact (Arnett et al., 2019). Be that as it may, there remains a considerable room for scientific inquiry in pursuit of novel and more refined avenues to prevent, diagnose, mitigate, and reverse different forms of cardiovascular ailment, as well as optimize patient management. Indeed, such a need for research in this field was even further emphasized as the world faced heightened health challenges during the COVID-19 pandemic with cardiovascular complications being among the most serious consequences of SARS-CoV-2 infection (Wehbe et al., 2020).

In the current Research Topic in Frontiers in Pharmacology, we aimed to shed light on the latest experimental techniques and methods used to investigate fundamental questions in Cardiovascular and Smooth Muscle Pharmacology. The topic features several articles that span the spectrum of cardiovascular research from natural product and drug action, including their underlying mechanisms in functional disorders of the heart and blood vessels, to mathematical and machine learning models for diagnosis and prediction of therapy outcomes of CVDs. The article assortment also includes discussion of cutting-edge research on diagnostic markers, therapeutic targets, and treatment technologies.

Indeed, natural product pharmacology received significant attention in this topic. Chen et al. explored the potential mechanism of polydatin glycosides on pulmonary hypertension by modulating endothelial-to-mesenchymal transition. In related studies, Zhao et al. and Deng et al. investigated the effect of astragaloside and nuciferine, the principal component of Lotus leaf extract, on the chronic intermittent hypoxia-induced endothelial dysfunction and endothelium-dependent vasodilation, respectively. Along the same lines, Shao et al. provide an in-depth discussion of the protective and therapeutic effect of Taohong Siwu on ischemic myocardial injury. On the other hand, small molecule drug pharmacology was also tackled in this issue, where Mustafa et al. review the available data describing the molecular pathways underlying the impact of sacubitril/valsartan on cardiac remodeling in heart failure. On the same premise, Yuan et al. propose gut microbiota as a target for the modulation of diabetic cardiomyopathy.

From a different perspective, Fares et al *.* offer new insight on the role mathematical processing of heart rate and blood pressure signals as predictors of early cardiovascular dysfunction associated with metabolic disease. Relatedly, in their study, Jia et al. explored the clinical implications and the sex differences in the role of circulating PCSK-9 in the development of atherosclerosis and as a biomarker for the severity of cardiovascular and metabolic disease. Significantly, the current topic is not lacking in contributions addressing advances in biomedical technology. Whereas Marei et al. highlight recent innovations in strategies for biofunctionalization of stents to reduce stent thrombosis in diabetes, Dai et al. explored the use of internet-based medical services and machine learning models to improve clinical outcomes of anticoagulant therapy. In the review article by Marei et al. *,* the authors discuss how thrombosis is one of the leading causes of stent failure in patients undergoing percutaneous coronary intervention (PCI). Stent thrombosis is primarily caused by impaired endothelialization of the stent lumen. Marei et al. discuss the mounting evidence supporting the use of circulating endothelial progenitor cells as a potential source for *in situ* endothelialization to prevent thrombosis and stent failure. This approach can have major implications in the treatment of coronary artery disease especially in type 2 diabetic patients who often presents with suboptimal outcomes following PCI or revascularization. Together, this research topic features novel developments in predicting cardiovascular dysfunction and provides insights into innovative interventions to alleviate cardiovascular insults that can pave the way for better diagnostic and therapeutic tools for CVDs.

## References

[B1] Arnett D. K. Blumenthal R. S. Albert M. A. Buroker A. B. Goldberger Z. D. Hahn E. J. (2019). 2019 ACC/AHA guideline on the primary prevention of cardiovascular disease: A report of the American college of cardiology/American heart association task force on clinical practice guidelines. Circulation 140 (11), e596–e646. 10.1161/CIR.0000000000000678 30879355 PMC7734661

[B2] Organization W. H. (2021). Cardiovascular diseases fact sheet. Available at: https://www.who.int/news-room/fact-sheets/detail/cardiovascular-diseases-(cvds)#:∼:text=Key%20facts,to%20heart%20attack%20and%20stroke .

[B3] Wehbe Z. Hammoud S. Soudani N. Zaraket H. El-Yazbi A. Eid A. H. (2020). Molecular insights into SARS COV-2 interaction with cardiovascular disease: Role of RAAS and MAPK signaling. Front. Pharmacol. 11, 836. 10.3389/fphar.2020.00836 32581799 PMC7283382

